# Patient and care characteristics of self-referrals treated by the general practitioner cooperative at emergency-care-access-points in the Netherlands

**DOI:** 10.1186/s12875-017-0633-1

**Published:** 2017-05-12

**Authors:** Martijn Rutten, Fieke Vrielink, Marleen Smits, Paul Giesen

**Affiliations:** Radboud Institute for Health Sciences, Scientific Center for Quality of Healthcare (IQ healthcare), Radboud University Medical Center, Geert Grooteplein 21, 6525 EZ Nijmegen, The Netherlands

**Keywords:** Primary health care, After-hours care, Referral, Gatekeeper, Health service, Efficiency, organizational, Emergency medical services

## Abstract

**Background:**

In the Netherlands, out-of-hours primary care is provided in general practitioner-cooperatives (GPCs). These are increasingly located on site and in collaboration with emergency departments of hospitals (ED). At such sites, also called emergency-care-access-points (ECAP), the GPC is generally responsible for the triage and treatment of self-referrals who used to attend the ED. To evaluate the effects and safety of this novel organisation, we studied the characteristics and the quality of care given by GPCs to self-referrals at ECAPs.

**Methods:**

Retrospective analysis (August 2011–January 2012) of 783 records of self-referred patients at three Dutch GPCs in an ECAP. This was supplemented with a retrospective analysis of patient records during a follow-up period of three-months to asses safety.

**Results:**

Patient-characteristics: 59% was male, 46% aged between 16–45 years and 59% trauma-related. Most cases (95%) were triaged low-urgent. None received the highest urgency-category. Quality: The triage outcome was correct in 79%, underestimated in 12% and overestimated in 9%. After GP consultation 20% were referred to the ED, mostly for radio-diagnostics. Of the referrals to secondary care, 98% were according to common medical practice. Thirty percent had a follow-up contact, mostly with their own general practitioner, seldom with the ED. Complications, all non-severe, were registered in 3.2%; 0.4% were possibly preventable.

**Conclusions:**

Self-referred patients at an ECAP are mostly trauma related, low-urgent and male patients. The majority could be treated by the GPC without subsequent referral to the ED. Care given at the GPC is reasonably efficient and safe. Triage and treatment of self-referrals by the GPC at ECAPs might offer opportunities for other countries facing problems with inappropriate emergency department visits.

## Background

The Netherlands have a strong primary care system. Most Dutch inhabitants have a general practitioner (GP), acting as a gatekeeper to hospital care. Patients who seek urgent medical care are however free to contact the primary care physician, call the emergency number (112), or visit the emergency department. Out-of-hours (emergency) primary care is provided in GP-cooperatives (GPCs) Table 1 [1, 2]. These are increasingly located on site and in collaboration with the accident and emergency departments (ED) of hospitals, forming an Emergency Care Access Point (ECAP) [3, 4]. At most of these co-located sites, the GPC is responsible for triage and treatment of self-referrals [4]. These patients were formerly able to consult the ED on their own initiative, without a referral of the GPC.

**Table 1:** Features of general practitioner cooperatives at emergency care access points in the Netherlands [1, 2, 4, 23]

Theme	Feature
General	Out-of-hours primary care is provided by large-scale general practitioner cooperatives (GPC)
	Out-of-hours is defined as daily from 5 p.m. to 8 a.m. the entire weekend, and public holidays.
	Participation of 50–250 GPs per cooperative with a mean of 4 h on call per week
	Population consists of 100,000 to 500,000 patients
	At present there are 121 GPCs with yearly about 4 million contacts. 200.000 self-referral contacts a year are registered at the GPCs (5%).
Location	Distance of patients to GPC maximally 30 km
	56% of GPCs is co-located with the ED of a hospital, forming an Emergency Care Access Point, 7% is located on the site of the hospital premises (without collaboration), 11% in the vicinity of the hospitals and 26% elsewhere
Accessibility	Access generally via regional telephone number. First contact is mostly telephonic with a triage nurse (90–95%), infrequently as self-referral.
	Telephone triage by nurses supervised by GPs: contacts are divided into telephone advice (38%), centre consult (52%), or GP home visit (10%).
	Triage outcomes (NTS: Dutch Triage Guidelines): Life threatening (U1) 2%; Acute (U2) 15%: Urgent (U3) 38%; Routine (U4) 18%; Advice (U5) 27%
	The GPC in an ECAP is mostly responsible for the face-to-face triage of self-referrals (54%). The ED is responsible for face-to-face triage in 21%. In 15% the triage is performed according to the patients choice. The remaining 10% has a deviant organisation.
	In the Netherlands, adult patients have to make an annual deductible (€385,- in 2016) for hospital care and diagnostics. GP and GPC care is fully covered, without a co-payment.
Facilities	Glucose testing and urine examination can be performed at all GPCs. An ECG is available in 26%, conventional radiology in 19% and routine laboratory test in 37–65%.

Self-referrals at the ED in the Netherlands are typically young men with trauma related complaints [5, 6]. They claim expensive specialist care and cause unnecessary attendance of the ED and longer waiting times [3, 6–10]. As in many Western countries, the Dutch EDs are increasingly struggling with overcrowding. Studies indicate that self-referred visits account for an average of 17% of all ED visits in the Netherlands, with a range of 3–58% [8, 10]. An estimated 51–80% of those self-referred patients could have been treated at the GPC, although the actual percentage has never been investigated [5, 11]. Due to increasing collaboration at ECAPs, these patients are now initially sent to the GPC, which is responsible for triage and subsequent treatment [4]. Recently, after establishing ECAPs a reduction in self-referral rates has been demonstrated at the ED [10, 12]. Although this substitution has a lot of social and political support in the Netherlands, concerns were expressed about the quality and safety of care provided by GPs especially in high-urgent emergencies. Therefore, we studied the quality of care given by GPCs to self-referrals at ECAPs.

## Methods

### Design and population

We carried out a retrospective patient record analysis of self-referrals attending the GPC of an ECAP. This was supplemented with a follow-up patient record study of three months at their own general practices. From August 2011 up until January 2012 we selected self-referrals from three GPCs in the Eastern part of the Netherlands. The selection procedure of self-referrals varied due to different sizes of ECAPS and logistic reasons. At the first GPC, all self-referrals during a two month period were selected (*n* = 295). At the second GPC, the researchers selected all self-referrals from a representative selection of seven participating GPs during a five month period (*n* = 301). Those GPs were chosen based on size of their practice, organisation and localisation. At the third GPC, the first sixty self-referrals of the month were selected, during a five month period (*n* = 300). Contacts were excluded in case of incomplete files or subsequent visits.

### Data collection

The data collection procedure consisted of four steps:

I. The medical records were extracted from the registration system of the GPC, providing information concerning the GPC visit. The researchers had access to the patients’ records of their own GP for the three subsequent months. This provided information about all possible contacts with healthcare workers (own GP, GPC, Out Patient Department Hospital, ED, ambulance emergency services, diagnostics).
II. The following routine variables were coded by one medical educated researcher:
*Patient characteristics:* gender, age, living area, urgency, eventual diagnosis (ICPC).
*Care characteristics:* diagnostics, treatment, referral to ED, reason for referral, subsequent advice.
*Follow-up*: follow-up contacts, complementary diagnostics, alterations in diagnostics or treatment by the GP or specialist, possible complications.

Subsequently, two medical educated researchers independently assessed a number of *subjective variables*:

- *Urgency:* adequacy of triage in retrospect, using the urgency categories of the Dutch NHG-Triage Index [13].
- *Guidelines:* applicability of guidelines formulated by the Dutch College of General Practitioners [14].
- *Clinical management:* appropriateness of the diagnostics, treatment and referral according to guidelines (if applicable) or common medical practice.
- *Adverse events:* unintended harm to the patient and preventability*.*

The assessment of the adequacy of triage was made in two of the three GPCs, because one GPC used a deviating triage tool.

III. The assessment of both researchers on the subjective variables were compared. As a next step, the researchers assessed the files in which they initially did not agree (panel 1).
IV. The cases without consensus after a discussion in step III were discussed with an experienced GP, as long as needed to reach consensus (panel 2).

Part of our data (mainly patient- and care characteristics) were used for a publication in a Dutch journal for General Practitioners [15].

### Ethics and privacy

The Ethical Research Committee of the Radboud university medical center Nijmegen was consulted and concluded that this study does not fall within the remit of the Dutch Medical Research Involving Human Subjects Act [Wet Mensgebonden Onderzoek]. All general practices gave their written permission for gathering and analysis of the patient records. To guarantee privacy, all researchers processed the data anonymously besides signing a declaration of confidentiality.

### Analyses

SPSS 20 (Statistical Package for Social Sciences) was used for data analyses. Study results were described using descriptive statistics and frequency tables.

## Results

### Inclusion and exclusion

In total 896 patients were selected. During analysis, 113 GPC contacts were excluded due to incomplete registration or subsequent visits at the GPC (16%). The remaining 783 records were further analysed.

### Agreement

The reviewers initially agreed on 543 (69%) of the medical records. In 240 (31%) records there was a discrepancy in one or more of the following variables: urgency, guideline applicability, clinical management and adverse events. After discussion between the two reviewers, there was consensus on 753 (96%) of all records. A discussion with the third reviewer resulted in consensus for all records.

### Objective characteristics

#### Patient characteristics

Table 2 shows the patient characteristics of the 783 self-referrals. Of these patients 59% was male. The mean age was 34.1 years; 46% was aged between 16 and 45 years. Their living area was mostly urban (65%). In 59% of the contacts, the patient had a trauma, most often a wound (24%), a suspicion of a fracture (16%) or another type of injury of the musculoskeletal system (13%). The diagnoses for self-referrals without trauma (39%) were rather varied. Of the 533 self-referrals assessed for adequacy of triage, 508 (95%) presented themselves with a lower urgency (level U3 or U4). The highest levels of urgency (U1) did not occur (Table 2). The patient characterises in the three GPCs were generally comparable

**Table 2:** Characteristics of the study population

Characteristic	Number	Percent
Gender (*n* = 783)		
• Male	459	(58.6)
• Female	324	(41.4)
Age (*n* = 783)		
• 0–15 years	184	(23.5)
• 16–45 years	361	(46.1)
• 46–65 years	150	(19.1)
• 65 > years	88	(11.2)
Living area (*n* = 783)		
• Urban	509	(65.0)
• rural area	274	(35.0)
Urgency (*n* = 533)^a,b^		
• U1: Life-threatening	0	(0)
• U2: Acute	25	(4.7)
• U3: Urgent	344	(64.5)
• U4: Routine	164	(30.8)
Diagnosis (ICPC) (*n* = 783)		
*Trauma*	*463*	*(59.1)*
• Wound	186	(23.8)
• suspicion of fracture of extremity	122	(15.6)
• contusion/distortion of musculoskeletal system	106	(13.5)
• multiple injury after trauma	26	(3.3)
• traumatic nose injury	14	(1.8)
• traumatic cranial injury	9	(1.1)
*Non trauma*	*320*	*(40.9)*
• abdominal complaints	41	(5.2)
• ocular complaints	34	(4.3)
• musculoskeletal (non-traumatic)	27	(3.4)
• myogenic complaints	20	(2.6)
• skin complaints	20	(2.6)
• thoracic pain	17	(2.2)
• respiratory complaints	14	(1.8)
• other	147	(18.8)

^a^Data refer to two out of three GPCs (second and third)

^b^Urgency U5 was introduced in the triage guidelines in 2012

#### Clinical management

In 580 cases (76%) the GPC was able to treat the self-referrals without referring. Twenty percent was referred to the ED after being seen by the GP. Only 4% was referred to the ED directly after triage (high urgency or need for diagnostics). The reason for referral in 102 cases (53%) concerned a request for X-ray diagnostics because of a suspected fracture (Table 3).

**Table 3:** Clinical Management at the general practitioner cooperative at first contact with a self-referred patient (*N* = 783)

Clinical management	Number		% total	% within category
Treatment by GPC	380		49	
Medication	156		20	
• analgesics		54	7	35
• oral antibiotics		32	4	20
• other		70	9	45
Suture, wound glue, skin closure	132		17	
• including tetanus toxoid or antibiotics		34	4	26
• excluding tetanus toxoid or antibiotics		98	13	74
Activity	92		12	
• bandage		48	6	52
• imprecisely defined (possibly with medication)		44	6	48
Other	11		1	
Conservative	200		26	
• explanation and advice		114	15	57
• wait and see (without explanation and advice mentioned)		86	11	43
Referral to ED	192		24	
Referral to the ED after GP consultation		157	20	82
Direct referral to the ED based on triage		35	4	18
Reason for referral (after GP consultation)				
• X-ray diagnostics needed	102		13	53
• specialist assessment needed	37		5	19
• acute assessment needed	10		1	5
• other	8		1	4
Total	783		100	

#### Follow-up

**Fig. 1:**
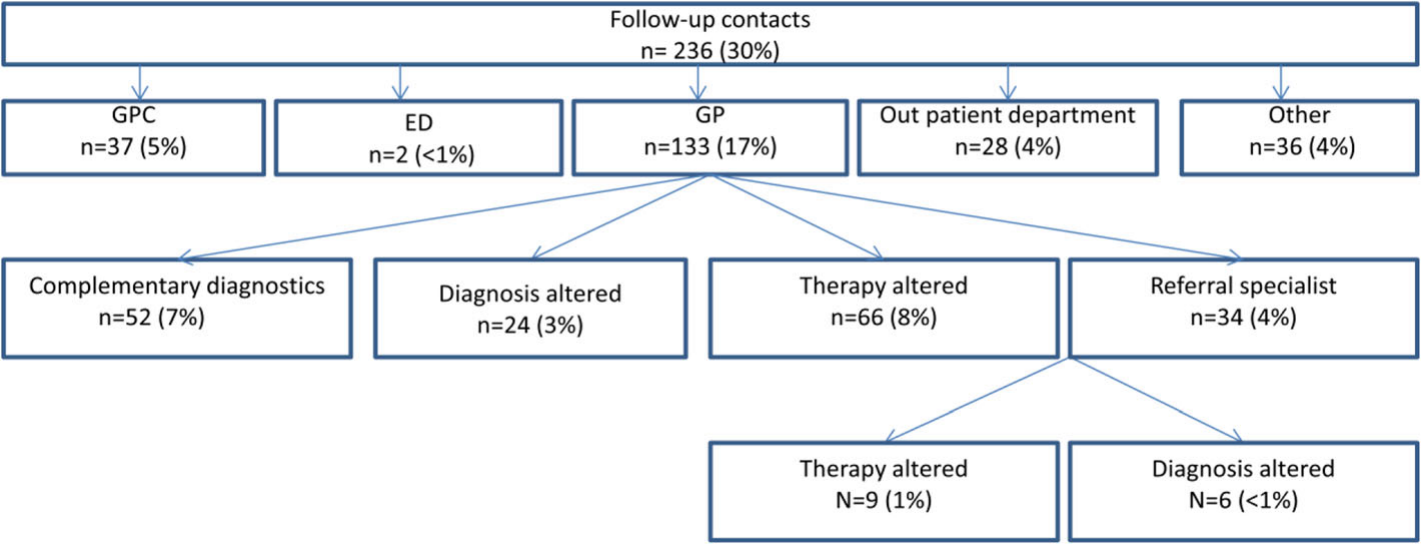
Follow-up contacts, diagnostics and treatment. Note: Percentages of total of 783 contacts. Multiple answers possible: for instance a General Practitioner could request complementary diagnostics and alter the diagnosis and/or treatment

Figure 1 provides an overview of the follow-up after the initial contact at the ECAP. Out of 783 self-referrals 236 (30%) had a follow-up contact. In 113 cases (17%) this concerned a contact with the patient’s own GP, in 37 cases (5%) the GPC. Only two self-referrals (0.3%) visited the ED, while the Dutch national emergency number 112 was never used. After the initial contact at the GPC the patient’s own GP performed complementary diagnostics in 52 patients (7%). In 24 cases (3%) the GP altered the diagnosis and in 66 cases (8%) the treatment was changed; 34 patients (4%) were referred to a specialist by their own GP.

### Subjective characteristics

#### Adequacy of triage

Table 4 shows the results of the judgment of the researchers about the correctness of the urgency level at triage. In 421 contacts (79%) the researchers judged in retrospect that the triage category was correct. In 63 contacts (12%) the urgency was underestimated by the triage nurses (undertriage) while in 49 cases (9%) it was overestimated (overtriage) (Table 4). Eighteen contacts (2%) were mistakenly judged by the triage nurses as being of lower-urgency (U3-U4), while in retrospect these turned out to be of the high-urgent (U1-U2) (not in table). However, this under-triage did not have any harmful consequences for the patients.

**Table 4:** Incorrect triage per urgency category

Urgency	Incorrect triage					
	Under triage		Over triage		Total	
	n	(%)	n	(%)	n	(%)
U1 Life-threatening (*n* = 0)	–		–		–	
U2 Acute (*n* = 25)	2	(8.0)	7	(28.0)	9	(36.0)
U3 Urgent (*n* = 344)	15	(4.4)	42	(12.2)	57	(16.6)
U4^a^Routine (*n* = 164)	46	(28.0)	-	-	46	(28.0)
Total^b^	63	(11.8)	49	(9.2)	112	(21.1)

^a^Overtriage is by definition not possible at the lowest level of urgency

^b^Data refer to two out of the three GPCs (second and third)

#### Guidelines

In 564 cases (72%) there was no guideline of the Dutch College of General Practitioners applicable according to the researchers. In 157 (72%) of the remaining 219 cases the GP adhered to the guidelines. The guidelines that were most often applicable were Injuries to the ankle ligament (19%), Red eye (15%), Traumatic knee problems (11%) and Acute coronary syndrome (7%).

#### Clinical management

According to the researchers, 619 self-referrals (79%) received the right type of diagnostics (anamnesis, physical examination, diagnostics). Inadequacies in recorded anamnesis or physical examination were predominantly the reason for cases in which the diagnostics were considered inadequate. Trauma screening to assess the risk of serious injury was often lacking during anamnesis.

In 666 cases (85%) the clinical management of the GP was considered appropriate. Predominant reasons for inappropriate clinical management (15% *n* = 117) were no or insufficient follow-up advice (51%), and no or incorrect type of medication prescription (38%). Of all referrals, 187 (98%) were judged correct.

#### Adverse events

None of the patients from the research population died or suffered from permanent adverse events. Complications related to GPC care occurred in 25 (3,2%) patients, of which three (0,4%) could have possibly been prevented (missed fracture hand, not recognized small arterial haemorrhage in a hand wound, infection due to poor dressing of a crush injury of a finger). Examples of unpreventable complications were delayed wound recovery and infection of the wound; these complications were related to their anatomical location or with co morbidity.

## Discussion

### Main findings

Insight into the quality of treatment of self-referrals at the GPC is highly relevant, due to the fact that the GPC and the ED have started working together more closely in ECAPs in the Netherlands. The GPC is increasingly responsible for the care provided for self-referrals, previously often provided by the ED. This collaboration contributes to reduce ED crowding. Patient characteristics and quality of care for self-referrals provided by the GPC at an ECAP have never been analysed.

Our study shows that care for self-referrals at the GPC is mostly low-urgent. Patients are often of young age, male and frequently present themselves with trauma. This corresponds with previous studies on self-referrals at the ED [3, 5, 6]. The triage performed by the GPC was considered correct in the majority of cases. However the urgency was underestimated in 12%, predominantly in the lowest urgency category (U4). Undertriage could generate potential harmful situations, although only 2% were mistakenly triaged as lower-urgent (U3-U4), while being high-urgent (U1-U2). The GPC is able to treat three quarters of all self-referrals, whereas a quarter was referred to the ED, mostly for conventional radio-diagnostics (53%). Almost all referrals to the ED were considered appropriate. In the majority of our cases the diagnostics applied and followed treatment were considered correct. Only one-third of all self-referrals had a follow-up contact, mostly with their own GP and seldom at the ED. Follow-up showed that a small number of patients suffered complications, none of these were serious adverse events. All these findings illustrate that a Dutch GPC is able to provide relative safe and effective care for self-referrals. In the Netherlands, as well as in many Western countries, emergency departments are overcrowding [16, 17]. The triage and treatment of self-referrals at an ECAP by the GPC should be considered as an efficient and presumably economical alternative for care at the ED. In this manner, the GP(C) maintains its role as a gatekeeper to hospital care [18, 19]. Besides, it can reduce overcrowding at ED, by limiting the patients inflow. The Dutch system might therefore offer opportunities for other countries facing problems with inappropriate emergency department visits.

### Strengths and weaknesses

One of the strengths of our study is that we did not only study contacts at the GPC, but also follow-up contacts for a three months period in other healthcare settings. This way we gained insight into the safety of care and eventual complications. Our study shows the actual percentage of referrals from GPC to ED and is not based on estimations as in earlier studies. The majority of the variables were based on objective data. The subjective variables were independently assessed and thoroughly analysed by two medically educated researchers. The assessments of both researchers on the subjective variables were compared and if necessary discussed. The researchers based their final judgment on guidelines, consensus discussions and expert consultation.

It is unclear whether the results can be generalised to other GPCs in the Netherlands or internationally. However, the results are generally the same for each of the three participating GPCs, which contributes to the generalisability. Nevertheless, population characteristics, local agreements and local customs in relation to care for self-referrals can differ considerably between GPCs, especially in metropolitan regions. Our study concerns a retrospective analysis of records, in which the issue of under-registration should be taken into account. The number of patients included in this study is limited, as a result no reliable conclusions could been drawn on the occurrence of adverse events and safety. Nevertheless this study gives some insight on safety of care for self referred patients by the GPC.

### Implications for practice and further research

The collaboration between a GPC and ED in ECAPs is successful, safe and efficient. The triage under responsibility of a GPC seems to be professional and safe. The percentage of potentially harmful under triage should however be reduced. This could be realised by offering training in face-to-face triage to triage nurses and adjustments and clarifications in the triage guidelines. GPs at a GPC provide efficient care, as only 20% of the patients were eventually referred to the ED. As a result, the ED could concentrate on providing high urgent complex emergency care, leading to a reduction in waiting and process times [3, 7–9, 18]. Although controversial in literature, a cost reduction is expected for this setting [8, 20]. Despite we did not find any adverse events in this study, further studies with larger patient numbers are advisable. Further studies on the effects of GPC care for self-referrals at other (metropolitan) ECAPs is recommended to assess the generalisability of the results.

Care for self-referrals in this setting consists mostly of trauma with a low-urgency. Increasingly GPCs are experimenting with nurse practitioners providing care for those patient categories. Positive outcomes are reported in the literature, both in daytime general practices and at GPCs, in terms of physicians’ workload, patients’ experiences, and care outcomes [21]. A study at one GPC showed that nurses can adequately deal with 77% of all consultations [22], but further studies on this subject are recommendable.

Further improvement in collaboration and efficiency could be realised by giving the GPC access to hospital diagnostic facilities (laboratory and conventional radiology). Currently, the diagnostics available for the GPC are limited and varied, only 19% has (restricted) access to conventional radiology [23]. Literature shows that GPs tend to use less resources compared to ED clinicians [9, 24]. The main reason for self referrals to attend an ED is an expected need for diagnostics [11, 17]. This study shows that 53% is referred by the GPC for conventional radio-diagnostics and less than half is expected to have an actual fracture [3]. By giving a GPC access to diagnostics, an additional reduction in referrals could be realised. Further research on those topics is recommended.

## Conclusion

Self-referrals at the ECAP are mostly young men. They frequently present with, trauma-related symptoms. Mostly it concerns low urgent care. The vast majority of self-referrals at the ECAP were treated by the GPC without subsequent referral to the ED. Treatment of self-referrals by the GPC should be considered as a safe, efficient and probably economical substitute for care at the ED and could help to reduce ED crowding.

## Abbreviations

ECAP: Emergency-care-access-points
ED: Emergency departments
GP: General practitioner
GPC: General practitioner-cooperative
